# The value of timely treatment with anti‐amyloid therapy for Alzheimer's disease

**DOI:** 10.1002/alz.71819

**Published:** 2026-10-07

**Authors:** Jack M. Chapel, Bryan Tysinger, Hossein Pourmand, Dana Goldman, Paul Aisen, for the Alzheimer's Disease Neuroimaging Initiative

**Affiliations:** ^1^ Sol Price School of Public Policy University of Southern California Los Angeles California USA; ^2^ Leonard D. Schaeffer Institute for Public Policy and Government Service University of Southern California Los Angeles California USA; ^3^ Alfred E. Mann School of Pharmacy and Pharmaceutical Sciences University of Southern California Los Angeles California USA; ^4^ Alzheimer's Therapeutic Research Institute University of Southern California San Diego California USA; ^5^ Keck School of Medicine University of Southern California Los Angeles California USA

**Keywords:** Alzheimer's disease, anti‐amyloid therapy, care pathway, donanemab, microsimulation, social value

## Abstract

**INTRODUCTION:**

Anti‐amyloid therapies can slow Alzheimer's disease (AD) progression; diagnostic and treatment delays might reduce their value.

**METHODS:**

Using dynamic microsimulation, we estimated the gross societal value of anti‐amyloid treatment for Americans with early symptomatic AD. A treatment scenario slowed the rate of clinical progression by 29% over individuals’ remaining lifetime (baseline mean age: 73.0); earlier treatment initiated 2 years earlier.

**RESULTS:**

Treatment generated $104,885 (95% CI: $99,291, $110,479) in gross societal value over an individual's lifetime, including quality‐of‐life gains, reduced caregiving burden, and medical cost offsets driven by reduced nursing home use and Medicaid spending. Earlier treatment increased value 32%; this rose to 71% if treatment effects are also stronger (36% slowing) in earlier AD stages. Scenarios with 4‐year effect duration yielded $49,122 (treatment) and $56,385 (earlier treatment).

**DISCUSSION:**

Timely treatment consistently increased long‐term societal value. The findings provide a benchmark against which treatment implementation costs can be weighed.

## Highlights

- Donanemab generates substantial societal benefits over an individual's lifetime.
- Earlier treatment initiation significantly increases long‐term value.
- Quality‐of‐life gains and reduced caregiver burden drive value.
- Medical cost offsets result from reduced nursing home use and Medicaid spending.

## 1 BACKGROUND

Alzheimer's disease (AD) imposes substantial societal costs due to high medical and long‐term care expenses, profound impacts on quality of life, and significant strains on care partners.^1^ The total economic costs of dementia in the United States reached an estimated $818 billion in 2026.^2^ Total costs are expected to grow as populations age and the number of AD cases continues to rise.^1^

Crucially, novel disease‐modifying therapies have emerged as promising tools to slow the progression of AD and mitigate the societal burden of disease.^3^, ^4^, ^5^ Two disease‐modifying anti‐amyloid therapies have received full approval from the US Food and Drug Administration (FDA): lecanemab in 2023 and donanemab in 2024. Both demonstrated an ability to clear amyloid plaques in the brain and slow the progression of cognitive and functional symptoms of AD.^3^, ^4^, ^6^, ^7^ However, concerns remain over the relative costs and benefits of available anti‐amyloid therapies^8^, ^9^, ^10^ and current system readiness for implementation, such as specialist supply, screening coverage, and infrastructure.^11^, ^12^, ^13^, ^14^ Decision makers must grapple with how to design coverage and financing strategies that will maximize the societal value of novel therapies while containing costs.

The timeliness of diagnosis and treatment initiation is a critical factor to consider when designing strategies to maximize the value of these therapies. On average, there is a 3.5‐year gap between perceived cognitive decline and official diagnosis^15^; the mean masks substantial heterogeneity by patient characteristics such as race and socioeconomic status.^16^, ^17^ Many individuals with probable dementia never receive a formal diagnosis at all.^18^, ^19^, ^20^, ^21^ The availability of disease‐modifying therapies increases the importance of timely diagnosis, since these therapies slow disease progression but do not reverse it. Delays in detection translate into delays in treatment initiation and continued disease progression during the intervening period, thereby reducing the potential to mitigate impacts through treatment. Moreover, there is suggestive evidence that treatments may be more effective when initiated in earlier stages of disease.^3^, ^7^, ^22^

In this study, we estimated the lifetime gross societal value per individual treated with anti‐amyloid therapy – the monetized health and economic benefits of treatment before netting out costs. We then quantified the potential gains in value if those individuals had received treatment 2 years earlier, but still after the onset of cognitive symptoms, consistent with improved detection and more timely treatment initiation. The 2‐year period was chosen to accommodate data constraints and because it represents a potentially achievable reduction in the current average 3.5‐year gap between symptoms and diagnosis.^15^ We used dynamic microsimulation modeling to project how observed clinical effects translate to impacts on health and economic outcomes over patients' remaining lifetimes and examined sensitivity to treatment effect magnitude and duration. We focused on estimating lifetime gross societal value and did not include costs of screening, treatment, or monitoring, which are likely to change as the diagnostics and treatment landscape continues to evolve. The resulting value estimates provide a benchmark against which implementation costs can be assessed and can inform the development of clinical care pathways, coverage policies, and investments in diagnostic infrastructure that promote patient access to timely treatment.

**RESEARCH IN CONTEXT**

1. **Systematic review**: The authors reviewed the literature using traditional sources (e.g., PubMed) and identified studies estimating the long‐term impacts of anti‐amyloid therapies. Several studies estimated the social value of anti‐amyloid therapies, but none quantified the added value of timely treatment initiation. These relevant citations are referenced.
2. **Interpretation**: Our findings provide evidence for decision makers designing coverage strategies and investing in diagnostic infrastructure. The estimated societal benefits from treatment initiated 2 years earlier demonstrate the importance of timely treatment initiation to maximize the value of anti‐amyloid therapy. The lifetime horizon reveals cost offsets concentrated in Medicaid through reduced long‐term care utilization.
3. **Future directions**: The microsimulation approach developed here can be extended to evaluate therapies with different efficacy profiles and treatment timing strategies. Future work could examine (a) the value of next‐generation treatments initiated in the presymptomatic stage and (b) how emerging blood‐based biomarker diagnostics affect the feasibility and societal impact of expanding screening strategies.

## 2 METHODS

### 2.1 Overview

We used dynamic microsimulation to extend the results of TRAILBLAZER‐ALZ 2, the pivotal phase 3 trial for donanemab,^3^ the most recent FDA‐approved anti‐amyloid therapy. The trial tested donanemab among adults aged 60 to 85 with early symptomatic AD, demonstrating a 29% slower increase in the Clinical Dementia Rating – Sum of Boxes (CDR‐SB) over 18 months compared to placebo.^3^ Donanemab is designed to be taken as a treatment course lasting 18 months or less, whereas the available alternative (lecanemab) is an ongoing treatment. A long‐term extension study found the slowing in disease progression continued through 36 months, including the 18 months after treatment discontinuation.^6^

We used a modified version of the US Cost of Dementia Model (USCDM)^2^, ^23^ – a dynamic microsimulation built from established and well‐validated predecessor models^24^, ^25^, ^26^ – to simulate the remaining life course of the clinical trial participants. We constructed a simulant cohort matching trial participants’ baseline characteristics and simulated their remaining life course under three scenarios: (1) *status quo* with no treatment; (2) *treatment*, representing donanemab administered as in the clinical trial; and (3) *earlier treatment*, where trial participants initiate treatment 2 years earlier than the trial. We compared individuals’ health and economic outcomes between scenarios to estimate the average lifetime societal value of treatment for this cohort, focusing in particular on the additional value generated by initiating treatment 2 years earlier.

### 2.2 Data sources

We used multiple data sources in the process of estimating the simulation model and constructing the simulant cohort to enter the model. Figure A1 summarizes how each data source contributes to the flow of estimation, cohort selection, and simulation.

Data primarily came from the Health and Retirement Study (HRS), 1998 to 2020. The HRS is a biennial, nationally representative longitudinal survey of Americans aged 51 and older, sponsored by the National Institute on Aging (grant number NIA U01AG009740) and conducted by the University of Michigan. We used data from the RAND HRS Longitudinal File, a version of the HRS data created by the RAND Center for the Study of Aging to facilitate analysis.^27^ The HRS collects detailed information on a wide range of health and economic topics, including diagnosed conditions, cognitive status, functional limitations, caregiving, nursing home entry, work and earnings, and mortality.

We also used data from the Alzheimer's Disease Neuroimaging Initiative (ADNI) database (adni.loni.usc.edu). The ADNI was launched in 2003 as a public‐private partnership, led by Principal Investigator Michael W. Weiner, MD. The primary goal of ADNI has been to test whether serial magnetic resonance imaging (MRI), positron emission tomography (PET), other biological markers, and clinical and neuropsychological assessment can be combined to measure the progression of mild cognitive impairment (MCI) and early Alzheimer's disease (AD). The ADNI data provided longitudinally observed CDR‐SB scores needed to estimate disease progression models, described below. Importantly, the ADNI data include measures of cerebrospinal fluid amyloid beta (Aβ) and phosphorylated tau at threonine 181 (p‐tau181), enabling us to estimate transition models specifically among an amyloid‐positive population that more closely reflects the clinical trial target population.

We used data from the Medicare Current Beneficiary Survey (MCBS), 2016 to 2019,^28^ to estimate medical expenditure models. The MCBS is a continuous, multipurpose survey of a nationally representative sample of the Medicare population, administered by the Centers for Medicare and Medicaid Services, that links survey‐reported data on health status, healthcare utilization, and access to care with Medicare claims data.

### 2.3 Key characteristics

This section describes the individual characteristics simulated in the microsimulation and how each was measured in our data.

We used the CDR‐SB^29^, ^30^ as the measure of clinical status. The CDR‐SB captures six domains, three cognitive (memory, orientation, and judgment/problem solving) and three functional (community affairs, home/hobbies, and personal care). Each domain is scored from 0 to 3, yielding a total score between 0 and 18. The CDR‐SB is a common endpoint used in trials of AD treatments; it was a secondary endpoint TRAILBLAZER‐ALZ 2 trial, with the integrated Alzheimer's Disease Rating Scale (not measured in HRS or ADNI) as the primary endpoint. In the simulation, we classified dementia severity by CDR‐SB score using validated ranges that correspond to global CDR stages^31^: 0 (unimpaired), 0.5 to 4.0 (MCI), 4.5 to 9.0 (mild dementia), 9.5 to 15.5 (moderate dementia), and 16.0 to 18.0 (severe dementia).

The HRS assesses cognition in the core survey, but it does not specifically include the CDR‐SB. However, CDR‐SB was part of the Aging, Demographics, and Memory Study (ADAMS), a substudy of the HRS in which in‐person clinical assessments for dementia were conducted for selected HRS respondents. We used the ADAMS sample to impute CDR‐SB in the rest of the HRS. Specifically, we used a matching approach in which each ADAMS respondent was matched (with replacement) to a core HRS respondent‐wave observation. Respondents were exactly matched on categorical dementia status (unimpaired, mild impairment, or dementia), age category, and proxy interview status and nearest‐neighbor matched on TICS27 score, number of activities of daily living (ADLs), number of instrumental IADLs (IADLs), years of education, and sex. Additional details are provided in Appendix B; Figure B1 shows the distribution of imputed CDR‐SB scores closely matches the observed distribution.

In brief, two data sources each contribute a distinct piece to how CDR‐SB enters the analysis. In ADNI, where CDR‐SB is directly and repeatedly assessed over time, we estimate how it progresses from one period to the next (Section 2.6) and characterize its trajectory in the 2 years before trial enrollment (Section 2.4). In the HRS, CDR‐SB is not typically collected, so we impute it from the ADAMS substudy. These imputed values are used to assemble a cohort resembling the trial population and to link CDR‐SB to downstream outcomes, but not to model its progression. In the simulation, each individual's CDR‐SB then advances over time according to the ADNI‐estimated progression model.

Other key measures included chronic diseases, functional limitations, unpaid caregiving receipt, nursing home residence, and work. Chronic conditions were based on self‐reported physician diagnoses and included including hypertension, diabetes, heart disease, lung disease, stroke, and cancer. Functional limitations included ADLs and IADLs, which capture difficulties with basic self‐care tasks and more complex household management tasks, respectively. Unpaid care hours captured the amount of help received from care partners, measured in hours per year. Work included paid employment or self‐employment.

### 2.4 Cohort selection

The primary objective of the study was to quantify the added value of treatment if clinical trial participants had initiated treatment 2 years earlier, which requires knowledge of participants’ CDR‐SB score before it was observed in the trial. To estimate trial participants’ recent trajectory of CDR‐SB, we constructed a matched ADNI cohort. We selected ADNI participants meeting the following criteria in their month 24 observation: age 60 to 85, had a CDR global score indicating MCI or mild dementia, and had presence of amyloid and tau pathology. Amyloid and tau pathology were defined based on cerebrospinal fluid measures of p‐tau181/Aβ42 ratio; those with p‐tau181/Aβ42 ≥ 0.0395 were selected based on published thresholds.^32^, ^33^ We used entropy balancing to reweight the ADNI cohort to match clinical trial participants’ baseline characteristics including age, sex, race, education, and CDR‐SB scores. Using the ADNI cohort, we estimated the mean CDR‐SB score 2 years earlier was 2.3 (compared to 3.9 at clinical trial baseline), and 96% of the cohort had a CDR global score greater than 0 (Table A1). This is consistent with the reported average onset of symptoms 4.1 years before the trial baseline in Clarity AD,^4^ the pivotal phase 3 trial for lecanemab; this information was not available for TRAILBLAZER‐ALZ 2.

We then constructed the USCDM cohort for the main analyses. We first selected HRS respondents meeting key inclusion criteria: aged 60 to 85 with MCI or mild dementia (CDR score of 0.5 or 1). Based on analyses from the ADNI cohort, we additionally required respondents to have a CDR global score greater than zero 2 years earlier. Consequently, all simulants had a global CDR score consistent with the donanemab indication at both the baseline and earlier treatment‐initiation times. We also excluded respondents with a history of non‐skin cancer within the past 5 years, a trial exclusion.

One‐third of the clinical trial participants were classified as having mild dementia at baseline, whereas only 13% of the potentially eligible HRS respondents with early symptomatic AD were classified as mild dementia. The clinical trial also significantly overrepresented highly educated and White populations. To increase the potential sample resembling clinical trial participants, we pooled HRS observations from 2010 to 2020 and selected respondents’ most recent observation in which they met the inclusion criteria. This yielded a final USCDM cohort of 6339 HRS respondents.

Clinical trial participants were highly selected on cognitive status, education, and race relative to the general population. Moreover, the HRS did not include information on amyloid pathology, and we could not directly select for amyloid positivity as required by the clinical trial. We therefore used a reweighting procedure^34^ to match the USCDM cohort to the clinical trial placebo group's baseline characteristics, including age, sex, race, education, CDR global score categories at baseline, and CDR‐SB scores at both baseline and 2 years earlier. Additional details are provided in Appendix C. We used these rebalancing weights in all subsequent analyses so that results were representative of the clinical trial participant population.

Because CDR‐SB is imputed for every HRS observation, each respondent has an imputed CDR‐SB 2 years before baseline (from their prior survey wave), which places them at the earlier point of their trajectory in the earlier treatment scenario. The matched ADNI cohort provided a trial‐representative target for CDR‐SB 2 years before baseline, which was not observable from the trial. The USCDM cohort's distribution of baseline and earlier CDR‐SB was then reweighted to match the ADNI cohort at both time points.

### 2.5 Dynamic microsimulation

USCDM is a dynamic microsimulation model that simulates the remaining life course of older Americans (age 51+).^2^, ^23^ It uses first‐order Markov transition models to simulate how individuals age, including onset of dementia and other chronic diseases, functional limitations, healthcare utilization and spending, caregiving, work status and earnings, and mortality. USCDM was developed based on the modeling framework of the Future Elderly Model,^24^ an established and well‐validated^25^, ^26^ microsimulation that has been used extensively in health and economics research, including serving as the analytic backbone for two National Academies reports^35^, ^36^ and several studies estimating long‐term consequences of AD prevention.^5^, ^37^, ^38^, ^39^, ^40^ Full technical details of the USCDM have been published elsewhere.^2^, ^23^ We made several modifications to the USCDM specifically for the present study. Here, we provide an overview of the model and describe extensions and modifications made specifically for the present study.

The microsimulation begins with the selected HRS cohort. At each 2‐year time step, the characteristics of each individual were updated based on predictions from the transition models in a Monte Carlo fashion. In each step, we calculated the dollar value of outcomes including caregiving hours, medical costs, earnings, health‐related quality of life, and caregiver disutility. Individuals who survived to the next period were carried forward to the subsequent time step, and the process continued until all simulated individuals had transitioned into the death state. We repeated the simulation of each scenario 100 times to produce stable estimates of the projected outcomes.

To account for uncertainty, we used a non‐parametric bootstrap to produce 95% confidence intervals (CIs) around the estimates. The HRS, ADNI, and MCBS data were each resampled 250 times, all models were re‐estimated for each sample, and the simulation was run 30 times for each of the 250 sets of re‐estimated models. Confidence intervals were then constructed as 1.96 times the standard deviation of the resulting bootstrap estimates. The bootstrap therefore reflects both sampling variability in the data and the estimation uncertainty of every model fit to it: the CDR‐SB progression model, the transition models for functional limitations, caregiving, nursing home residence, work, and mortality, the medical cost models, and the health‐related quality‐of‐life model. Parameters that were imposed rather than estimated from the data, including the treatment‐effect magnitude and duration, were instead examined through one‐way sensitivity analyses. The reported confidence intervals should therefore be interpreted as conditional on the imposed assumptions.

### 2.6 Transition models

The CDR‐SB transition model was estimated using ADNI data among participants with probable amyloid and tau pathology at baseline, defined as having a p‐tau181/Aβ42 ratio ≥ 0.0395 based on thresholds from Hansson et al.^32^ and Smith et al.^33^ The model was estimated using ordinary least squares regression with current CDR‐SB as the outcome variable and covariates including a linear spline of 2‐year lagged CDR‐SB (with a knot at 4.5), age, sex, and race. The spline specification allows the rate of CDR‐SB progression to differ at higher levels of baseline impairment. Model coefficients are provided in Table A2.

We estimated this transition model in ADNI rather than HRS for two reasons. First, the HRS does not include direct, longitudinal CDR‐SB measurements; CDR‐SB is imputed cross‐sectionally from ADAMS (Section 2.3), and we did not estimate dynamic transition models on imputed values, to avoid propagating imputation error into the projected disease trajectory. ADNI, by contrast, provides the repeated direct CDR‐SB assessments needed to model period‐to‐period progression. Second, the ADNI biomarker measures allow us to restrict the estimation sample to amyloid‐ and tau‐positive individuals resembling the trial population; they are not covariates in the model. Because the transition model's covariates (prior CDR‐SB, age, sex, and race) are all observed in the HRS, the estimated progression relationship can be applied directly to the HRS simulation cohort. Amyloid positivity in the simulation cohort is instead addressed through cohort selection and reweighting to the trial population (Section 2.4).

In the simulation, changes in CDR‐SB directly affected transition models of ADLs, IADLs, informal caregiving hours received, nursing home entry, and work status, all of which were estimated using HRS data. These transition models included CDR‐SB along with other covariates such as lagged outcome measures, age, sex, marital status, race/ethnicity, education, and diagnosed chronic conditions. Nursing home entry and work status were estimated using probit models, and ADLs and IADLs used ordered probit models. Informal caregiving hours were modeled in a multi‐stage process: any receipt of informal care, receiving full‐time (24 h per day) caregiving conditional on receiving any care, and hours of care conditional on receiving care but not full‐time care. CDR‐SB affected mortality indirectly through its impact on ADLs, IADLs, and nursing home residency status. Because greater functional limitation and nursing home residence independently predict higher mortality in these models, slowing CDR‐SB progression may indirectly lower mortality risk and extend life expectancy. These transition models are shown in Tables A3 and A4.

### 2.7 Valuation of outcomes

We assigned dollar values to the following outcomes in each simulation step, drawing both on transitioned states from the simulation and on cross‐sectional models applied to the simulated characteristics. Earnings were a transitioned outcome in the simulation and depended on work status, which was directly impacted by CDR‐SB. Informal caregiver hours were valued at $34 per hour. Caregiver disutility impacts were estimated based on the number of caregivers and stage of dementia, using estimates from Lin et al.^41^

Medical costs were estimated based on simulated health characteristics, functional limitations, and nursing home status in each period, using models estimated from MCBS data. These models capture total medical spending from all sources, including patient and family out‐of‐pocket payments. Total costs were also disaggregated into Medicare, Medicaid, and other payers, which combines out‐of‐pocket payments with private and supplemental insurance. Medical cost models are shown in Table A5.

Health‐related quality of life was also assigned in each simulation step based on current health, cognition, and function. This assignment used a cross‐sectional model estimated among a subset of HRS respondents that participated in a module that included the Health Utilities Index Mark 3 (HUI3) instrument (Table A6). We then valued changes in health‐related quality of life using the Generalized Risk‐Adjusted Cost‐Effectiveness (GRACE) framework.^42^, ^43^, ^44^ GRACE generalizes traditional cost‐effectiveness analysis by incorporating two features of preferences that standard approaches omit: the diminishing marginal utility of health and the higher willingness to pay for health improvements among individuals in worse health states. Under standard cost‐effectiveness analysis, a quality‐adjusted life year (QALYs) is assigned the same dollar value regardless of the recipient's baseline health; GRACE relaxes this assumption by allowing the marginal value of a QALY to vary with illness severity. We implemented GRACE using a constant relative risk aversion constant relative risk aversion utility function over health. This specification implies that health improvements are more valuable to individuals with lower baseline health, consistent with stated‐preference evidence on the severity‐adjusted value of health gains. This approach is particularly relevant for AD therapies, where treated individuals have substantially impaired quality of life and traditional cost‐effectiveness methods may understate the value of health improvements. Table A7 shows results using HUI3 compared to GRACE to assess how the method of health utility valuation influences results.

### 2.8 Simulation scenarios

We simulated three scenarios:

1. Status quo, in which treatment is never initiated;
2. treatment, in which treatment is initiated at the clinical trial baseline (indexed as *t* = 0); and
3. earlier treatment, in which treatment is initiated 2 years before the clinical trial (indexed as *t* = −2).

All simulations began at *t* = −2, when the cohort mean age was 71.

To calculate the lifetime social value per person treated for each scenario, we compared the stream of outcomes in each treatment scenario to the status quo; the difference in outcomes between the treatment and earlier treatment scenarios represented the value of more timely treatment. We refer to the sum of these differences in monetized outcomes as the *gross societal value* of treatment. It is “gross” in that it does not net out the costs of treatment, screening, biomarker testing, amyloid‐related imaging abnormalities (ARIA) monitoring, or infusion administration. All scenarios were discounted to a common reference point at the start of the simulation (*t* = −2, 2 years before the trial baseline), using a 3% annual discount rate. All monetary figures were adjusted to 2025 US dollars using the Personal Consumption Expenditure Price Index.

### 2.9 Treatment effects

Treatment effects were derived from the TRAILBLAZER‐ALZ 2 clinical trial results. Treatment was modeled as a 29% slowing of CDR‐SB progression, consistent with the observed effect of donanemab relative to placebo in the combined population at 76 weeks.^3^ Specifically, in each 2‐year simulation cycle the modeled increment in CDR‐SB (i.e., predicted by the transition model relative to the previous period) was multiplied by 0.71 for treated individuals, so that CDR‐SB advanced at 71% of the untreated rate each cycle. The treatment effect was applied only to increases in CDR‐SB and did not lower CDR‐SB below its prior level. Predicted CDR‐SB was capped at the 0 to 18 range of the scale.

We conducted sensitivity analyses to evaluate the robustness to alternative treatment parameter assumptions. We varied the magnitude of the treatment effect from 10% to 100% slowing of CDR‐SB progression, holding duration at lifetime. While the upper end of this range (100% slowing, i.e., complete halting of CDR‐SB progression) is not achievable with currently available therapies, it serves as a benchmark bracketing the total burden of disease in the cohort. These analyses also provide insight into the value of potential next‐generation therapies with different efficacy profiles.

### 2.10 Persistence of treatment effects

The treatment effect magnitude is taken directly from the pivotal clinical trial results, but the duration of these effects beyond the trial period is not yet known. In our primary analysis, we assumed the treatment effect (i.e., the rate of slowing in CDR‐SB progression) persists for the patient's remaining lifetime, consistent with a disease‐modifying effect. This assumption reflects the proposed mechanism of amyloid removal: Rather than conferring a direct symptomatic benefit on cognition or clinical status, these therapies remove accumulated amyloid – the initiating event of the disease^45^, ^46^, ^47^ and, through the early symptomatic stages, a driver of its progression.

Consistent with this mechanism, the pivotal trials of both successful amyloid removing therapies – donanemab^3^ and lecanemab^4^ – demonstrate a change in the slope of clinical decline. Evidence from the trials is suggestive that the benefit of donanemab is greater when treatment is initiated at earlier disease stages,^3^, ^6^, ^22^ consistent with amyloid driving progression early in the disease course, before amyloid‐independent processes increasingly contribute to decline. A large trial of a third amyloid‐targeting therapy, solanezumab, did not meet clinical endpoints, but this may be because it did not clear amyloid plaques, consistent with plaque removal underlying the effect.^48^

Direct evidence on the persistence of this change in slope far beyond the trial period will remain limited until longer‐term data become available. However, evidence from studies extending the follow‐up of trial participants up to 3 years provides some suggestive evidence of persistence of benefit after treatment is discontinued. Donanemab is administered as a limited‐duration course, and most early‐start participants in TRAILBLAZER‐ALZ 2 completed treatment after reaching amyloid clearance and received no further active dosing; their benefit nonetheless appeared to be retained through 3 years, while cleared amyloid reaccumulated only slowly.^6^ Because this comparison is against an external control cohort rather than a randomized placebo group, it is suggestive rather than definitive. The lecanemab phase 2 trial offers a complementary off‐treatment observation, with the benefit accrued during dosing maintained across an average 2‐year gap after discontinuation.^49^ These observations extend only a few years beyond treatment, however, leaving the longer‐term trajectory unobserved.

Because of this, we did not treat lifetime persistence as a certainty. In sensitivity analyses, we examined this uncertainty by varying the duration over which the rate of slowing is applied from 2 years through lifetime treatment. The lower bound of this range corresponds to a purely symptomatic effect in which the benefit does not persist beyond active treatment.

### 2.11 Adverse events

We incorporated adverse events into the simulation based on rates observed in the TRAILBLAZER‐ALZ 2 trial. We followed the Institute for Clinical and Economic Review and other similar modeling studies and included effects of symptomatic ARIA with edema/effusion (ARIA‐E) and treatment discontinuation due to adverse events.^41^, ^50^, ^51^ Other adverse events, such as asymptomatic ARIA, infusion‐related reactions, and treatment‐related death, were not included but would likely have a minimal effect on our results: Asymptomatic ARIA carries no symptom burden, infusion‐related reactions are predominantly mild and transient, and treatment‐related death is rare.^3^, ^6^, ^7^ Symptomatic ARIA‐E occurred in approximately 6% of donanemab‐treated participants,^3^ which we modeled as a 12‐week period of reduced quality of life for a random 6% of the treated cohort. Additionally, 13% of treated individuals were modeled as discontinuing treatment due to adverse events, resulting in no treatment effect for those individuals. These adverse events were included at the same rate in all scenarios, including sensitivity analyses, in which individuals receive treatment.

## 3 RESULTS

The USCDM cohort included 6339 HRS respondents (Table 1). After reweighting, the USCDM cohort closely matched the trial population on key characteristics at baseline. Mean age at baseline (*t* = 0) was 73.0 years, 43% were male, 91% were non‐Hispanic White, 61% had a global CDR score of 0.5 (MCI), and 39% had a score of 1 (mild dementia). Mean baseline CDR‐SB was 3.9, identical to the trial population. The estimated mean CDR‐SB 2 years prior to baseline (*t* = −2) was 2.3, reflecting the progression of cognitive impairment leading up to trial enrollment. One‐third of the cohort had activity limitations at *t* = 0 and 25% received any unpaid care. Only 17% were working for pay in *t* = 0, reflecting the older age of the cohort.

**TABLE 1:** Summary of USCDM cohort compared to clinical trial participants.

	HRS respondents		Clinical trial participants^b^
Characteristic	Base weight	Reweighted	
*N*	6339	6339	1736
Age^a^	72.3	73.0	73.0
(SD)	(7.9)	(7.1)	(6.2)
Male^a^	0.51	0.43	0.43
Race			
White^a^	0.76	0.91	0.92
Black	0.16	0.07	0.02
Other race	0.09	0.02	0.06
Hispanic ethnicity	0.13	0.15	0.06^c^
13+ years of education^a^	0.29	0.72	0.72
CDR global score			
0.5^a^	0.87	0.61	0.61
1	0.13	0.39	0.36
2	0.00	0.00	0.03
CDR‐SB^a^	2.1	3.9	3.9
(SD)	(1.7)	(2.3)	(2.1)
CDR‐SB, 2‐year lag^a^	1.8	2.3	2.3^d^
ADL limitations			
No limitations	0.72	0.64	
1 ADL	0.13	0.14	
2 ADLs	0.06	0.08	
3+ ADLs	0.10	0.14	
IADL limitations			
No limitations	0.73	0.62	
1 IADL	0.13	0.13	
2+ IADLs	0.14	0.26	
Quality‐adjusted life year (HUI3)	0.66	0.61	
Working for pay	0.21	0.17	
Any unpaid caregiving received	0.16	0.25	
Lives in a nursing home	0.02	0.03	

Abbreviations: HRS, Health and Retirement Study; CDR, Clinical Dementia Rating; CDR‐SB, CDR Sum of Boxes score; ADL, activities of daily living; IADL, instrumental activities of daily living. The “Base weight” column uses the core HRS respondent weights; the “Reweighted” column uses the weights constructed to match the clinical trial population baseline characteristics.

^a^ Matching variable for reweighting.

^b^ Baseline characteristics reported in TRAILBLAZER‐ALZ 2 study^4^ among combined population; statistics displayed combine the treatment and placebo groups.

^c^ Hispanic ethnicity is only reported for the subpopulation of US participants in the trial; race is reported for all trial participants.

^d^ The 2‐year lag of CDR‐SB among clinical trial participants is estimated from a matched ADNI cohort.

Figure 1 displays the trajectory of CDR‐SB scores under the status quo and treatment scenarios and compares it to the placebo and treatment groups in the TRAILBLAZER‐ALZ 2 trial. The mean score among the USCDM cohort closely tracked the observed groups’ mean during the trial period. Beyond the trial period, cognitive decline continued as expected: Simulated mean CDR‐SB (among survivors) increased to approximately 8.4 at *t* = 4 years and 12.4 for those surviving to *t* = 8 years.

**FIGURE 1:**
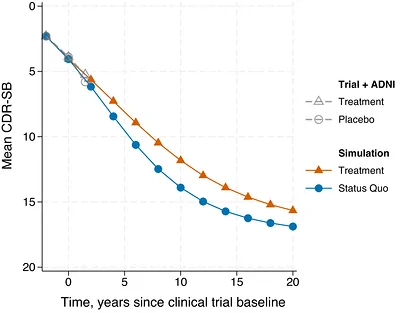
Trajectory of CDR‐SB in the USCDM simulation compared to the TRAILBLAZER‐ALZ 2 trial. Lines show the mean CDR‐SB score among surviving cohort members under the simulation status quo and treatment scenarios, alongside the trial placebo and donanemab arms. Over the trial period, the simulated status quo trajectory tracks the trial placebo arm and the simulated treatment trajectory tracks the trial donanemab arm, indicating that the model reproduces both the untreated trajectory and the treatment effect. Time is measured in years since the clinical trial baseline. The clinical trial trajectory between *t* = −2 and *t* = 0 is estimated based on a matched ADNI cohort (Table A1).

In the status quo scenario, individuals lived an additional 12.5 years since *t* = −2 (i.e., 2 years before the clinical trial), when the cohort was aged 71 on average (Figure 2). They spent an average of 3.9 years in the MCI stage, 2.4 years in mild dementia, 2.5 years in moderate dementia, and 3.7 years in severe dementia over their remaining lifetimes. Treatment shifted the distribution toward less severe disease stages: Individuals spent 0.5 more years with MCI, 0.5 more years with mild dementia, 0.3 more years with moderate dementia, and 1 less year with severe dementia. Note that these differences reflect a shift in the proportion of time spent in each dementia stage as well as a 0.3‐year extension in total life years lived. Earlier treatment further amplified these benefits, with nearly 0.9 more years spent with MCI, 0.5 more years with mild dementia, 0.2 more years with moderate dementia, and 1.2 fewer years with severe dementia, relative to status quo. In other words, treatment extended longevity while significantly increasing the proportion of life years lived in less severe stages of dementia.

**FIGURE 2:**
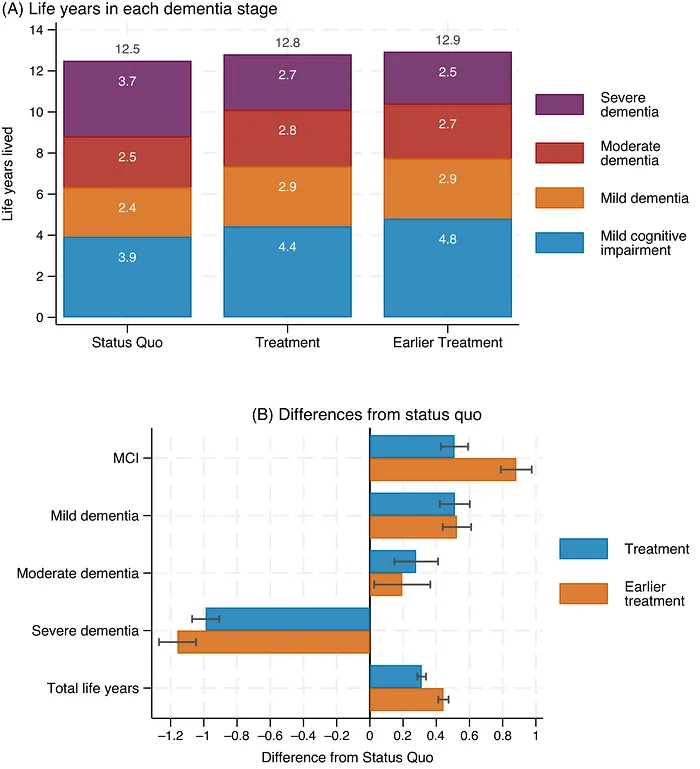
Average life years lived in each dementia stage. (A) Total years lived, starting from a baseline mean age of 71, under status quo and each treatment scenario; total life years are shown above each bar. (B) Difference in life years lived in each dementia stage under each treatment scenario relative to status quo, with bootstrapped 95% confidence intervals. Dementia stages are based on simulated CDR‐SB in each period: MCI is 0.5 to 4.0, mild dementia is 4.5 to 9.0, moderate dementia is 9.5 to 15.5, and severe dementia is 16.0 to 18.0.

Table 2 summarizes the projected remaining lifetime outcomes under each scenario. Under the status quo scenario, individuals lived an average of 12.5 years (95% CI: 12.2, 12.8), accumulating 6.9 (6.6, 7.2) QALYs, 3.6 (3.4, 3.8) disability‐free years, 10.0 (9.5, 10.5) years living in the community, and 1.3 (1.2, 1.4) years working for pay. Treatment increased total life expectancy by 0.31 (0.28, 0.34) years, QALYs by 0.46 (0.44, 0.48), disability‐free years by 0.37 (0.34, 0.40), years in the community by 0.63 (0.59, 0.67), and working years by 0.05 (0.04, 0.06). Earlier treatment generated larger gains relative to status quo across all outcomes: 0.44 (0.41, 0.47) additional life years, 0.62 (0.59, 0.65) additional QALYs, 0.57 (0.54, 0.60) additional disability‐free years, 0.83 (0.78, 0.88) additional years in the community, and 0.08 (0.07, 0.09) additional working years.

**TABLE 2:** Impact of treatment scenarios on lifetime outcomes.

	Life years lived since *t* = −2 (mean age 71)				
Scenario	Total	Quality‐adjusted^a^	Disability‐free^b^	In community	Working for pay
Status quo, levels	12.5	6.9	3.6	10.0	1.3
	(12.2, 12.8)	(6.6, 7.2)	(3.4, 3.8)	(9.5, 10.5)	(1.2, 1.4)
Differences from status quo					
Treatment	0.31	0.46	0.37	0.63	0.05
	(0.28, 0.34)	(0.44, 0.48)	(0.34, 0.40)	(0.59, 0.67)	(0.04, 0.06)
Earlier Treatment	0.44	0.62	0.57	0.83	0.08
	(0.41, 0.47)	(0.59, 0.65)	(0.54, 0.60)	(0.78, 0.88)	(0.07, 0.09)

*Note*: Bootstrapped 95% confidence intervals are in parentheses. All simulations start at *t* = −2, when cohort was age 71 on average.

^a^ Generalized risk‐adjusted quality‐adjusted life years (GRA‐QALY) calculated using a constant relative risk‐aversion utility‐of‐health function.

^b^ Disability‐free life‐years were defined as years lived in the community (i.e., not in a nursing home) without limitations in activities of daily living (ADL) or instrumental ADLs.

These impacts translated to substantial lifetime societal value (Table 3). Treatment initiated at the clinical trial baseline generated a total lifetime societal value of $104,885 (95% CI: $99,291, $110,479) per person. The largest component of value was the increase in health‐related quality of life for the person living with AD, which was valued at $52,339 ($50,313, $54,365) using the GRACE framework. Reductions in unpaid caregiving hours contributed $23,783 ($21,047, $26,519), and gains in caregivers’ quality of life added $5829 ($5415, $6243). Medical cost offsets totaled $22,177 ($18,696, $25,658). Increased patient earnings contributed $757 ($621, $893), reflecting the low rate of working at baseline. Earlier treatment increased total value to $138,256 ($129,903, $146,609) per person, representing a 32% increase relative to the treatment scenario. The additional value from earlier treatment derived from larger gains across all components.

**TABLE 3:** Value of lifetime benefits from treatment.

	Lifetime value per person (2025 USD)	
Value compoment	Treatment	Earlier treatment
Gain in patient quality of life^a^	52,339	72,714
	(50,313, 54,365)	(69,729, 75,699)
Gain in patient earnings	757	1276
	(621, 893)	(1075, 1477)
Medical cost offsets	22,177	26,286
	(18,696, 25,658)	(21,719, 30,853)
Reduction in unpaid care hours^b^	23,783	30,498
	(21,047, 26,519)	(26,934, 34,062)
Gain in unpaid care partner quality of life^c^	5829	7483
	(5415, 6243)	(6921, 8045)
Total social benefit	104,885	138,256
	(99,291, 110,479)	(129,903, 146,609)

*Note*: Bootstrapped 95% confidence intervals are in parentheses. Lifetime benefits per person are the present value of all future outcomes discounted to the year of treatment initiation, calculated using a 3% discount rate. Values are adjusted to 2025 dollars using the Personal Consumption Expenditure Index.

^a^ Quality‐of‐life gains valued using the Generalized Risk Adjusted Cost Effectiveness framework.

^b^ Unpaid care hours are valued at $34/hour.

^c^ Unpaid care partner disutility depends on number of caregivers and stage of dementia, using estimates from Lin et al. (2023).41

The treatment scenarios generated medical cost offsets despite the increase in longevity. Figure 3 shows these cost offsets disaggregated by payer and annualized to account for the differences in longevity. Under the status quo, average annual medical costs totaled $63,583, including $32,001 of Medicare spending and $12,398 of Medicaid spending. Treatment generated $2527 in annual cost offsets, with Medicaid offsets accounting for $1638. Importantly, these medical cost figures do not account for the costs of treatment. Earlier treatment generated larger offsets, reducing total costs by $3292 per year, $2080 of which came from Medicaid spending. Medicaid accounted for a disproportionate share of cost offsets relative to its share of total spending, reflecting the concentration of long‐term care and nursing home costs in the Medicaid program.^52^ Compared to the status quo, annual Medicaid costs were 13% lower under the treatment scenario and 17% lower under the earlier treatment scenario.

**FIGURE 3:**
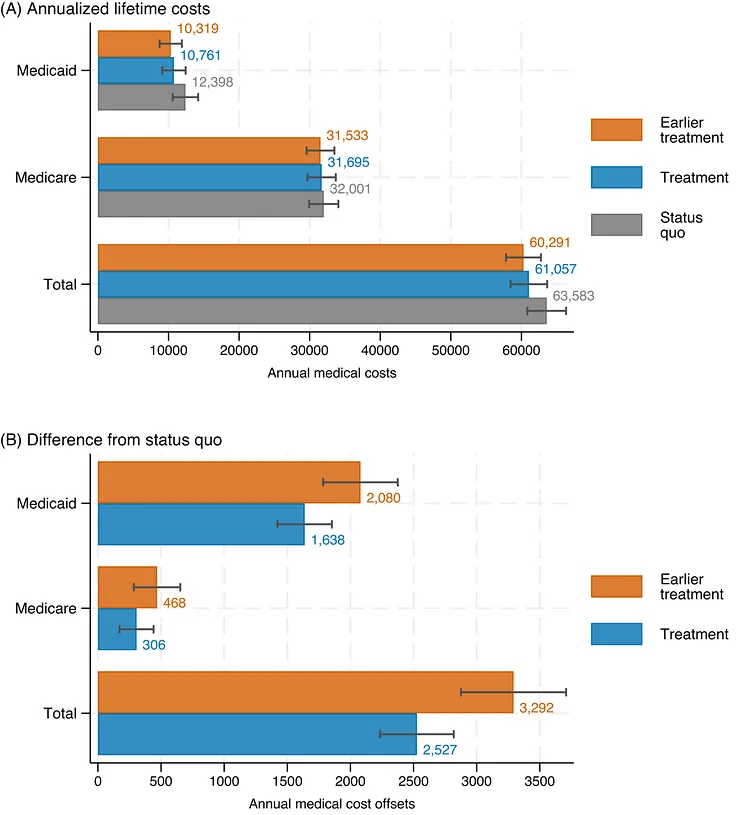
Impact of treatment scenarios on annual medical costs. (A) Annualized total, Medicare, and Medicaid costs per person in each scenario. (B) Difference in annualized costs between treatment scenarios and status quo. Spikes represent bootstrapped 95% confidence intervals. Annualized medical costs are defined as total remaining lifetime costs divided by life years lived. Costs do not include costs of treatment, screening, or monitoring.

The estimated impacts of treatment depend heavily on assumed treatment effect parameters. We assessed alternative assumptions about the rate of slowing of CDR‐SB progression and the persistence of this effect (Figure 4). If treatment achieved 50% slowing of progression, the lifetime societal value would increase to $208,670 in the treatment scenario and $276,688 for earlier treatment; at 100% slowing – complete halting of progression, which can be interpreted as a benchmark representing total burden of disease – values would reach $529,695 and $648,300. Accounting for the statistical uncertainty in the trial‐reported treatment effect (95% CI: 18.3% to 39.5% slowing), lifetime gross societal value ranged from $61,616 to $153,801 for in the treatment scenario and from $81,419 to $201,770 under the earlier treatment scenario (Table A8).

**FIGURE 4:**
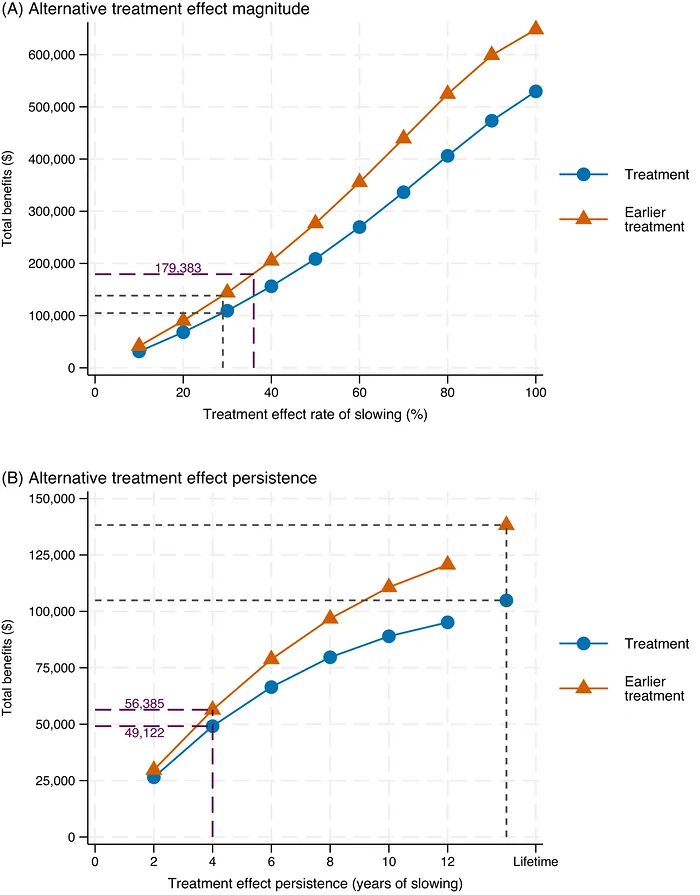
Gross societal value of treatment with varying treatment effect parameters. (A) Gross societal value of treatment in scenarios varying treatment effect magnitude (rate of slowing in CDR‐SB increase), with lifetime treatment duration. The gray dotted lines indicate value from 29% slowing in the main analyses; the purple dashed line indicates the value corresponding to 36% slowing in earlier treatment, consistent with effects observed for the low/medium tau group in the clinical trial.^3^ (B) Gross societal value of treatment in scenarios varying treatment effect persistence, defined as duration of years the 29% rate of slowing in CDR‐SB increase is applied. The gray dotted lines indicate the value from lifetime 29% slowing in main analyses; the purple dashed lines indicate the value corresponding to 4 years of 29% slowing.

Varying the duration over which the rate of slowing was applied also had a substantial impact on the societal value of treatment. In more conservative scenarios with a 4‐year effect duration instead of lifetime, the societal value was $49,122 (treatment) and $56,385 (earlier treatment). Across all of these assumptions, earlier treatment generated meaningfully more gross societal value than treatment initiated at the trial baseline, with the relative gain larger when the effect was assumed to persist longer.

## 4 DISCUSSION

This study estimated the lifetime societal value of anti‐amyloid therapy for individuals with early symptomatic AD and quantified the additional value of initiating treatment 2 years earlier, consistent with improved detection and more timely diagnosis. We found that donanemab treatment initiated at the clinical trial baseline generates $104,885 in lifetime societal value per person; initiating the same treatment 2 years earlier increases this value to $138,256 – a 32% gain. Benefits accrued across multiple domains, led by gains in patient quality of life, reduced caregiving burden, and medical cost offsets. Both treatment scenarios reduced time spent in severe dementia by more than a year.

The study's findings build on a growing body of evidence assessing the long‐term value of disease‐modifying therapies for AD.^5^, ^41^, ^50^, ^53^, ^54^ For example, Tysinger and colleagues valued the 10‐year gross societal value of lecanemab at $38,000 to $95,000 per person treated,^5^ and Tahami Monfared et al. estimated the annual societal value per person treated was $19,710 to $37,351.^53^ Similarly, Boustani et al. used a value‐based pricing framework to estimate an “economically justifiable price” for donanemab of $91,126 for the societal perspective.^50^ Differences in modeling frameworks, efficacy assumptions, time horizons, and data sources contribute to this variation, underscoring the importance of comprehensive outcome assessment and methodological transparency.^51^, ^54^ Our estimated 0.3‐year life extension in the treatment scenario is similar to or lower than projected life expectancy impacts in similar studies, which ranged from approximately 0.3 to over 1 year.^50^, ^51^, ^53^, ^54^, ^55^, ^56^

The persistence of treatment benefits is a central uncertainty in our analysis. Other economic evaluations have similarly found durability after discontinuation to be a primary driver of value.^50^, ^51^, ^54^ Our primary estimates assume the slowing in CDR‐SB progression persists over the remaining lifetime, consistent with a disease‐modifying mechanism, similar to the limited‐duration donanemab model of Boustani et al.^50^ If the effect instead persisted only 4 years, lifetime societal value would fall to $49,122 under the treatment scenario and $56,385 under the earlier treatment scenario (Figure 4B). Earlier treatment nonetheless generated more value than trial‐baseline treatment under every duration we examined, with the added value growing larger under longer effect duration.

A central contribution is quantifying the cost of diagnostic delay now that disease‐modifying therapies are available. The 32% higher lifetime societal value (or potentially as high as 71%) from treating 2 years earlier represents value recoverable by reducing care‐pathway delays. A recent meta‐analysis found an average of 3.5 years between symptom onset and dementia diagnosis,^15^ up to 4.5 years in US studies.^16^, ^17^, ^57^ In Clarity AD, the pivotal lecanemab trial, there was a nearly 3‐year gap between participants’ reported symptom duration and time since diagnosis.^4^ Moreover, many individuals with probable dementia never receive a formal diagnosis.^18^, ^19^, ^20^, ^21^ Disparities in time to diagnosis by race and socioeconomic status further compound this problem.^16^, ^17^

Earlier treatment was projected to generate more value because it is initiated at a lower level of impairment (average CDR‐SB of 2.3 vs 3.9), affording a greater range over which to benefit from slowed progression. Some evidence suggests that treatment may be more efficacious when initiated earlier, at lower accumulated amyloid,^3^, ^6^, ^7^ consistent with the amyloid cascade hypothesis.^45^ If treatment effects are indeed larger at earlier disease stages, the added value of timely treatment would substantially exceed our main estimates. For example, if earlier treatment achieved 36% slowing (rather than 29%), as reported for the low/medium tau group in TRAILBLAZER‐ALZ 2,^3^ it would generate $179,383, a 71% increase over the treatment scenario initiated 2 years later.

Realizing these benefits requires overcoming substantial barriers beyond diagnostic technology. Establishing eligibility typically requires multiple specialist visits and confirmatory amyloid testing, entailing travel and wait times that vary with uneven regional access to PET imaging and dementia specialists.^11^, ^12^, ^58^ Treatment then imposes recurring costs, including monthly or biweekly infusions at unevenly distributed centers, periodic MRI monitoring for ARIA, and adverse‐event management.^59^ Out‐of‐pocket costs under Medicare Part B, limited provider supply, and disparities by geography, race, and socioeconomic status may further constrain uptake and widen inequities in dementia care.^16^, ^17^ The value we estimate will be realized only to the extent that the care pathway is made accessible and equitable.

These findings underscore the importance of investments to improve the dementia care pathway. Emerging blood‐based biomarker tests, particularly for plasma p‐tau‐217,^60^, ^61^ can accurately detect amyloid pathology and may enable earlier identification of treatment‐eligible individuals in primary care; the first such test was cleared by the FDA in 2025,^62^ and primary care physicians appear open to adopting them.^63^ Complementary strategies, such as brief cognitive assessments,^64^ digital detection tools,^65^ streamlined referral pathways, and public awareness campaigns, could accelerate the path from symptom recognition to treatment.^66^ Ongoing trials of presymptomatic anti‐amyloid treatments^67^, ^68^ could further increase benefits if successful, potentially increasing the importance of early detection.

The composition of value highlights domains that are frequently overlooked in narrow cost‐effectiveness analyses. Quality‐of‐life gains were the largest component ($52,339 under the treatment scenario and $72,714 under earlier treatment), reflecting the fundamental purpose of disease‐modifying therapies: preserving cognitive function, independence, and quality of life. Reductions in informal caregiving burden were the second‐largest contributor ($23,783 and $30,498), capturing both the substantial time costs borne by family members and the quality‐of‐life impacts of caregiving. Earnings gains were relatively modest ($757 and $1276), reflecting the older age of the treated population with low baseline employment; impacts in younger‐onset populations might be larger. Medical cost offsets were $22,177 and $26,286, approaching but not fully covering the current drug cost ($32,000 for 12 months^69^). This is consistent with the view that the merits of treatment should not rest solely on cost savings, given the significant societal value from other sources.

The distribution of medical cost offsets across payers has notable policy implications. Medicaid accounted for a disproportionate 65% of cost offsets. This pattern reflects the concentration of long‐term care costs in the Medicaid program.^52^ Treatment extends time living in the community by 0.63 to 0.83 years; even modest delays in institutionalization generate substantial savings given annual nursing home costs exceeded $110,000 in 2024.^70^, ^71^ Medicare savings were more modest ($306 to $468 per year), as Medicare primarily covers acute care and physician services that are less directly affected by dementia stage. This distribution represents a potential misalignment: Treatment costs are borne primarily by the federal government through Medicare, while substantial downstream savings accrue to states (as well as the federal government) through Medicaid. Beyond the public payers, other payers were the second‐largest source of cost offsets. Because this category includes patient and family out‐of‐pocket payments, treatment may ease the financial burden borne directly by families; however, our cost models did not distinguish out‐of‐pocket spending from private and supplemental insurance.

Our estimates are best interpreted as a benchmark of the gross societal value of treatment against which the costs of implementation can be weighed. The total value accrued over patients’ remaining lifetime ($104,885 and $138,256) substantially exceeds the current list price for a 12‐month course of treatment (approximately $32,000^69^); this price may fall as competition increases and alternative disease‐targeted therapies enter the market.^72^ These estimates exclude costs of screening, biomarker testing, ARIA monitoring, or infusion administration; Arbanas and colleagues, for example, estimated $5864 in annualized Medicare administration costs per lecanemab patient beyond the drug itself.^10^ The considerably higher value under earlier treatment reinforces the benefits of investments in diagnostic infrastructure that enable earlier detection and treatment initiation.

### 4.1 Limitations

Our study was subject to several limitations. First, our main estimates assumed that the treatment effect persists for the patient's remaining lifetime (as expected for a disease‐modifying intervention). While evidence from the long‐term extension trial showed persistence through 36 months,^6^ the trajectory beyond this period is uncertain; our sensitivity analyses examine this uncertainty. Second, clinical trial participants were highly selected relative to the general AD population: They were predominantly non‐Hispanic White (92%) and had high levels of education. Our estimates therefore are not necessarily generalizable to broader populations. Third, CDR‐SB was imputed in the HRS rather than directly observed, and amyloid status could not be confirmed, introducing measurement error. Relatedly, the medical cost models were estimated in the MCBS and applied to the simulated cohort; although we defined cost predictors consistently across surveys, residual differences in measurement could introduce error into predicted costs. Finally, we did not include the costs of treatment administration, screening, or monitoring; our gross societal value estimate should be weighed against these implementation costs, which are likely to change as competition increases and new modes of treatment (e.g., at‐home subcutaneous administration) become available.

## 5 CONCLUSION

Anti‐amyloid therapy for early symptomatic AD can generate substantial societal value over patients’ remaining lifetimes, and timely diagnosis and treatment initiation significantly increase value. The current average diagnostic delay of 3.5 years represents a recoverable loss of value, and because value accrues broadly across patients, care partners, and payers, investments in earlier detection may be warranted. These estimates serve as a benchmark to be weighed against the costs of treatment, screening, and monitoring not captured here.

## AUTHOR CONTRIBUTIONS

**Alzheimer's Disease Neuroimaging initiative**: Data used in preparation of this article were obtained from the Alzheimer’s Disease Neuroimaging Initiative (ADNI) database (adni.loni.usc.edu). As such, the investigators within the ADNI contributed to the design and implementation of ADNI and/or provided data but did not participate in analysis or writing of this report. A complete listing of ADNI investigators can be found at: http://adni.loni.usc.edu/wp‐content/uploads/how_to_apply/ADNI_Acknowledgement_List.pdf.

## CONFLICT OF INTEREST STATEMENT

All authors are affiliated with the USC Schaeffer Institute, which receives funding from a wide variety of public and private institutions and donors, including Eli Lilly and Company. Dr. Chapel reports research grants from the National Institute on Aging, the National Bureau of Economic Research, Alzheimer's Association, Genentech, and the Society of Actuaries. Dr. Tysinger reports research grants from the National Institute on Aging. In the last 3 years, Dr. Goldman reports grants from American Heart Association, Alexion, Amgen, Biomarin, Blue Cross Blue Shield of Arizona, Blue Cross Blue Shield of Massachusetts, BrightFocus, Bristol Myers Squibb, California Hospital Association, Cedars‐Sinai Health System, Charles Koch Foundation, CommonSpirit, Edwards Lifesciences, Gates Ventures, Genentech, Gilead Sciences, Incyte, Johnson & Johnson, Lilly, National Institute on Aging, National Institute of Diabetes and Digestive and Kidney Diseases, Novartis, Pfizer, RA Capital, and Sarepta Therapeutics; personal fees from Edwards Lifesciences, and GRAIL. He is a co‐founder and scientific advisor of EntityRisk and holds equity in the company. Dr. Aisen has research grants from NIH, the Alzheimer's Association, Lilly and Eisai, and consults with Merck, Roche, Genentech, AbbVie, Biogen, Arrowhead, ImmunoBrain Checkpoint, AltPep, Bristol Myers Squibb, Johnson&Johnson and New Amsterdam. Author disclosures are available in the Supporting Information.

## CONSENT STATEMENT

This study used de‐identified secondary data from the Health and Retirement Study (HRS), the ADNI, and the Medicare Current Beneficiary Survey (MCBS). The HRS was approved by the University of Michigan Institutional Review Board, and all participants provided written informed consent. The ADNI was approved by the Institutional Review Boards of all participating sites, and written informed consent was obtained from all participants. The MCBS is authorized by Section 1875 of the Social Security Act and conducted by NORC at the University of Chicago with protections under the Privacy Act of 1974. No direct interaction with human subjects was conducted for the present analysis, and additional informed consent was not required.

## Supporting information



Supporting Information

Supporting Information
