# Supplementary material for: The value of timely treatment with anti‐amyloid therapy for Alzheimer's disease
Source: Alzheimers Dement. 2026 Oct 7;22(10):e71819. doi: 10.1002/alz.71819 (PMC13643942; doi:10.1002/alz.71819)
Supplement: Supplementary file 2 [file ALZ-22-e71819-s002.docx]

**APPENDIX**

Contents

[Appendix A. Supplemental Tables and Figures	2](#_Toc233495861)

[Figure A1. Summary of data and methods	2](#_Toc233495862)

[Table A1. Summary of ADNI cohort matching clinical trial population for two-year lookback	3](#_Toc233495863)

[Table A2. CDR-SB transition model	4](#_Toc233495864)

[Table A3. Functional limitations, work, and nursing home residence models	5](#_Toc233495865)

[Table A4. Help hours models	7](#_Toc233495866)

[Table A5. Medical costs models	9](#_Toc233495867)

[Table A6. Health-related quality of life model	13](#_Toc233495868)

[Table A7. Health-related quality of life using alternative methods	14](#_Toc233495869)

[Table A8. Range of gross societal value of treatment accounting for trial-reported uncertainty	15](#_Toc233495870)

[Appendix B. Imputation of CDR-SB in the HRS	16](#_Toc233495871)

[Figure B1. Distribution of CDR-SB in imputed and observed samples	17](#_Toc233495872)

[Appendix C. Construction of analysis cohort	18](#_Toc233495873)

[Sample Selection	18](#_Toc233495874)

[Reweighting	18](#_Toc233495875)

# Appendix A. Supplemental Tables and Figures

### Figure A1. Summary of data and methods

Abbreviations: HRS, Health and Retirement Study; ADNI, Alzheimer's Disease Neuroimaging Initiative; MCBS, Medicare Current Beneficiary Survey; CDR-SB, Clinical Dementia Rating–Sum of Boxes; GRA-QALY, Generalized Risk Adjusted Quality Adjusted Life Year.

### Table A1. Summary of ADNI cohort matching clinical trial population for two-year lookback

|  | ADNI cohort | |
| --- | --- | --- |
|  | Unweighted | Reweighted |
| N | 208 | 208 |
| Age | 74.9 | 73.0 |
| (SD) | (5.0) | (6.2) |
| White | 0.94 | 0.92 |
| Educated 13+ years | 0.86 | 0.72 |
| CDR 0.5 | 0.68 | 0.61 |
| CDR 1 | 0.32 | 0.39 |
| CDR-SB | 3.5 | 3.9 |
| (SD) | (2.1) | (2.1) |
| CDR-SB, 2 years earlier | 2.1 | 2.3 |
| (SD) | (1.3) | (1.3) |
| CDR 0.5+, 2 years earlier | 0.94 | 0.96 |

Notes: CDR, Clinical Dementia Rating; CDR-SB, CDR – Sum of Boxes; ADNI, Alzheimer's Disease Neuroimaging Initiative. ADNI cohort includes participants observed in month 24 with valid cerebrospinal fluid Aβ42 and pTau181 measurements. The reweighted column uses weights from entropy balancing to match the clinical trial population baseline characteristics (shown in Table 1 in the text). Entropy balancing matched the mean and variance of age and CDR-SB, and the mean of White, educated 13+ years, and CDR 0.5.

### Table A2. CDR-SB transition model

|  | CDR-SB |
| --- | --- |
| 2-year lag of CDR-SB spline, <4.5 | 1.650*** |
|  | (0.069) |
| 2-year lag of CDR-SB spline, 4.5+ | 1.016*** |
|  | (0.077) |
| Age spline, <85 | 0.025 |
|  | (0.014) |
| Age spline, 85+ | -0.087 |
|  | (0.100) |
| Male | -0.031 |
|  | (0.183) |
| White | 0.686 |
|  | (0.441) |
| Intercept | -1.948 |
|  | (1.152) |
| R^2^ | 0.626 |
| N | 765 |

Abbreviations: CDR-SB, Clinical Dementia Rating – Sum of Boxes; ADNI, Alzheimer's Disease Neuroimaging Initiative; OLS, ordinary least squares.

Notes: OLS regression model with current CDR-SB as the dependent variable. Estimated among ADNI participants with cerebrospinal fluid pTau181/Aβ42 ratio ≥ 0.0395, indicating probable amyloid and tau pathology, based on thresholds from Hansson et al. (2018) and Smith et al. (2025). The CDR-SB lag variable uses a linear spline specification with a knot at 4.5, allowing the rate of CDR-SB progression to differ at higher levels of baseline impairment. Age uses a linear spline with a knot at 85. Standard errors are in parentheses. *p < 0.05, **p < 0.01, ***p < 0.001.

### Table A3. Functional limitations, work, and nursing home residence models

|  | ADLs (ordered probit) | IADLs (ordered probit) | Working for pay (probit) | Nursing home (probit) |
| --- | --- | --- | --- | --- |
| CDR-SB | 0.069*** | 0.140*** | -0.030*** | 0.097*** |
|  | (0.001) | (0.002) | (0.003) | (0.002) |
| Two-year lag of Has exactly 1 ADL | 1.000*** | 0.441*** |  |  |
|  | (0.012) | (0.015) |  |  |
| Two-year lag of Has exactly 2 ADLs | 1.415*** | 0.621*** |  |  |
|  | (0.018) | (0.020) |  |  |
| Two-year lag of Has 3 or more ADLs | 1.944*** | 0.787*** |  |  |
|  | (0.018) | (0.019) |  |  |
| Two-year lag of Has exactly 1 IADL | 0.371*** | 0.944*** |  |  |
|  | (0.014) | (0.014) |  |  |
| Two-year lag of Has 2 or more IADLs | 0.513*** | 1.548*** |  |  |
|  | (0.017) | (0.018) |  |  |
| Two-year lag of R working for pay |  |  | 2.178*** |  |
|  |  |  | (0.011) |  |
| Two-year lag of nursing home status |  |  |  | 2.459*** |
|  |  |  |  | (0.042) |
| Non-Hispanic Black | 0.118*** | 0.039* | 0.043* | -0.247*** |
|  | (0.015) | (0.016) | (0.019) | (0.041) |
| Hispanic | 0.174*** | 0.112*** | -0.028 | -0.465*** |
|  | (0.019) | (0.021) | (0.025) | (0.064) |
| Less than high school | 0.084*** | 0.095*** | -0.092*** | 0.046 |
|  | (0.015) | (0.016) | (0.022) | (0.035) |
| Some college and above | -0.057*** | -0.073*** | 0.106*** | -0.110** |
|  | (0.013) | (0.015) | (0.015) | (0.034) |
| Male | -0.046** | -0.014 | 0.169*** | -0.022 |
|  | (0.016) | (0.017) | (0.019) | (0.041) |
| Male × Less than high school | -0.005 | 0.059* | 0.035 | -0.051 |
|  | (0.023) | (0.025) | (0.032) | (0.058) |
| Male × Non-Hispanic Black | -0.027 | 0.052* | -0.126*** | 0.371*** |
|  | (0.024) | (0.026) | (0.029) | (0.064) |
| Male × Hispanic | -0.059* | -0.027 | 0.000 | 0.306** |
|  | (0.029) | (0.032) | (0.035) | (0.099) |
| Male × Some college and above | -0.004 | -0.043 | 0.015 | 0.005 |
|  | (0.021) | (0.023) | (0.023) | (0.055) |
| Age spline, <65 | -0.007* | -0.016*** | -0.074*** | 0.016 |
|  | (0.003) | (0.003) | (0.003) | (0.013) |
| Age spline, 65-74 | 0.009*** | 0.014*** | -0.022*** | 0.027*** |
|  | (0.002) | (0.002) | (0.002) | (0.005) |
| Age spline, 75+ | 0.024*** | 0.029*** | -0.053*** | 0.034*** |
|  | (0.001) | (0.001) | (0.003) | (0.002) |
| Log(years since previous interview) | 0.204*** | 0.171*** | -0.238*** | 0.533*** |
|  | (0.025) | (0.028) | (0.030) | (0.066) |
| Heart disease | 0.145*** | 0.162*** | -0.101*** | 0.053* |
|  | (0.010) | (0.011) | (0.013) | (0.024) |
| Stroke | 0.304*** | 0.319*** | -0.265*** | 0.363*** |
|  | (0.013) | (0.014) | (0.022) | (0.026) |
| Cancer | 0.089*** | 0.080*** | -0.079*** | -0.018 |
|  | (0.011) | (0.012) | (0.015) | (0.028) |
| Hypertension | 0.085*** | 0.075*** | -0.103*** | -0.012 |
|  | (0.010) | (0.011) | (0.011) | (0.025) |
| Diabetes | 0.152*** | 0.116*** | -0.081*** | 0.148*** |
|  | (0.010) | (0.011) | (0.013) | (0.026) |
| Lung disease | 0.253*** | 0.267*** | -0.206*** | 0.077* |
|  | (0.013) | (0.014) | (0.019) | (0.032) |
| Widowed | 0.052*** | 0.031* | -0.010 | 0.225*** |
|  | (0.011) | (0.012) | (0.015) | (0.026) |
| Cut 1 | 1.409 | 1.078 |  |  |
|  | (0.190) | (0.211) |  |  |
| Cut 2 | 2.025 | 1.77 |  |  |
|  | (0.190) | (0.211) |  |  |
| Cut 3 | 2.464 |  |  |  |
|  | (0.190) |  |  |  |
| Intercept |  |  | 3.364*** | -4.415*** |
|  |  |  | (0.184) | (0.806) |
| Pseudo R^2^ | 0.286 | 0.364 | 0.541 | 0.534 |
| N | 136,989 | 136,989 | 136,989 | 136,989 |

Abbreviations: ADL, activity of daily living; IADL, instrumental ADL; CDR-SB, Clinical Dementia Rating – Sum of Boxes.

Notes: Models estimated using HRS data, 1998–2020. ADL and IADL models are ordered probit specifications with categories: 0, 1, 2, and 3+ limitations (ADLs) and 0, 1, and 2+ limitations (IADLs). Working for pay and nursing home residence are probit models. All models include the two-year lag of the respective outcome variable, CDR-SB, demographic characteristics, chronic conditions, and the log of years (measured with months) since the previous interview to account for variation in interview timing. Cut points are thresholds for the ordered probit models. Standard errors are in parentheses. *p < 0.05, **p < 0.01, ***p < 0.001.

### Table A4. Help hours models

|  | Help hours per year | | |
| --- | --- | --- | --- |
|  | Any hours  (probit) | Max hours (probit) | Number of hours (OLS) |
| Total help-hours received/year (10s), 2-year lag | 0.003*** | 0.001*** | 3.259*** |
|  | (0.000) | (0.000) | (0.070) |
| CDR-SB | 0.091*** | 0.033*** | 65.071*** |
|  | (0.002) | (0.003) | (2.408) |
| Age spline, <65 | -0.015*** | -0.023 | 2.841 |
|  | (0.003) | (0.020) | (7.991) |
| Age spline, 65-74 | 0.003* | 0.031*** | 24.517*** |
|  | (0.002) | (0.009) | (3.986) |
| Age spline, 75+ | 0.038*** | 0.003 | 7.293** |
|  | (0.001) | (0.004) | (2.444) |
| Male | -0.079*** | -0.057 | -44.728 |
|  | (0.009) | (0.045) | (23.091) |
| Married | -0.084*** | 0.199** | 326.152*** |
|  | (0.012) | (0.070) | (31.185) |
| Non-Hispanic Black | 0.218*** | 0.230*** | 73.293* |
|  | (0.012) | (0.051) | (28.586) |
| Hispanic | 0.323*** | 0.150* | 50.982 |
|  | (0.015) | (0.063) | (34.569) |
| Less than high school | 0.062*** | 0.157** | 411.424*** |
|  | (0.012) | (0.050) | (27.604) |
| Some college and above | 0.052*** | -0.001 | -221.014*** |
|  | (0.010) | (0.053) | (25.312) |
| Heart disease | 0.160*** | 0.068 | 176.657*** |
|  | (0.010) | (0.042) | (23.066) |
| Stroke | 0.344*** | 0.230*** | 351.432*** |
|  | (0.014) | (0.043) | (27.569) |
| Cancer | 0.109*** | 0.117* | 105.666*** |
|  | (0.011) | (0.047) | (26.793) |
| Hypertension | 0.129*** | 0.011 | -44.054 |
|  | (0.009) | (0.049) | (24.199) |
| Diabetes | 0.240*** | 0.008 | 43.430 |
|  | (0.010) | (0.044) | (23.665) |
| Lung disease | 0.342*** | 0.114* | 212.978*** |
|  | (0.013) | (0.051) | (29.042) |
| Widowed | -0.141*** | 0.089 | 141.449*** |
|  | (0.015) | (0.074) | (35.396) |
| Intercept | -0.389* | -1.604 | -752.521 |
|  | (0.177) | (1.191) | (483.324) |
| Pseudo R^2^ | 0.164 | 0.145 |  |
| R^2^ |  |  | 0.195 |
| N | 126,616 | 28,941 | 28,441 |

Abbreviations: CDR-SB, Clinical Dementia Rating – Sum of Boxes; OLS, ordinary least squares.

Notes: Help hours models estimated using HRS data, 1998–2020. Help hours are modeled in a multi-stage process: (1) a probit model for any receipt of informal care; (2) a probit model for receiving maximum (full-time, 24 hours per day) caregiving, conditional on receiving any care; and (3) an OLS model of hours of care per year, conditional on receiving care but not full-time care. The two-year lag of total help hours received per year is measured in tens of hours. Standard errors are in parentheses. *p < 0.05, **p < 0.01, ***p < 0.001.

### Table A5. Medical costs models

|  | Annual medical cost | | | |
| --- | --- | --- | --- | --- |
|  | Total | Medicare, Part A | Medicare, Part B | Medicaid |
| Age 65-69 | -8,567*** | -4,150 | -4,425 | -1,531 |
|  | (2,550) | (2,642) | (2,947) | (1,513) |
| Age 70-74 | -7,471** | -3,468 | -4,301 | -1,701 |
|  | (2,547) | (2,648) | (2,945) | (1,520) |
| Age 75-79 | -5,706* | -3,345 | -3,921 | -1,260 |
|  | (2,555) | (2,647) | (2,950) | (1,514) |
| Age 80-84 | -5,460* | -2,987 | -3,807 | -1,050 |
|  | (2,553) | (2,654) | (2,947) | (1,543) |
| Age 85+ | -5,537* | -2,667 | -4,706 | -1,028 |
|  | (2,560) | (2,661) | (2,947) | (1,550) |
| Male | 297 | 663 | -466 | -1,275** |
|  | (673) | (410) | (394) | (477) |
| Male × Black | 231 | -1,456 | 2,077* | 1,256* |
|  | (1,335) | (1,074) | (982) | (559) |
| Male × Hispanic | 1,521 | 1,806 | -1,403 | 1,369* |
|  | (1,883) | (1,444) | (1,019) | (638) |
| Male × Less than HS | -740 | -1,360 | -203 | -1,016 |
|  | (1,184) | (860) | (633) | (622) |
| Male × College and above | 94 | -299 | 780 | 1,670** |
|  | (820) | (481) | (471) | (584) |
| Black | 1,302 | 2,225** | 392 | -148 |
|  | (794) | (809) | (461) | (341) |
| Hispanic | 2,299** | -138 | 2,654** | -726* |
|  | (827) | (610) | (813) | (345) |
| Less than high school | 1,025 | 1,568** | 58 | 823 |
|  | (687) | (594) | (395) | (433) |
| Some college and above | 787 | 73 | -18 | 9 |
|  | (442) | (256) | (267) | (345) |
| Widowed | -1 | 116 | 14 | -1,184** |
|  | (414) | (276) | (245) | (403) |
| Single | 1,203* | 246 | 132 | -1,278** |
|  | (513) | (318) | (302) | (392) |
| Diagnosis stage for Cancer | 17,488*** | 4,062** | 9,845*** | 397 |
|  | (2,203) | (1,297) | (1,402) | (920) |
| Diagnosis stage for Diabetes | -1,071 | -1,475 | -22 | -2,406** |
|  | (1,229) | (787) | (640) | (842) |
| Diagnosis stage for Hypertension | 1,166 | 1,688* | 615 | 213 |
|  | (923) | (720) | (428) | (869) |
| Diagnosis stage for Heart disease | 6,337*** | 1,887* | 2,884** | -334 |
|  | (1,620) | (902) | (1,024) | (991) |
| Diagnosis stage for Lung disease | 8,271** | 2,588* | 1,564 | -521 |
|  | (2,814) | (1,203) | (1,332) | (753) |
| Diagnosis stage for Stroke | 14,585*** | 8,098*** | 4,162*** | 2,543 |
|  | (2,580) | (2,060) | (1,104) | (1,605) |
| Diagnosis stage for Heart attack | 8,590** | 7,739** | 1,451 | 189 |
|  | (2,869) | (2,587) | (1,306) | (1,446) |
| Diagnosis stage for Dementia | 3,351* | 3,492* | 1,508 | -1,552 |
|  | (1,654) | (1,364) | (891) | (1,043) |
| Maintenance stage for Cancer | 5,663*** | 501* | 3,090*** | -58 |
|  | (582) | (247) | (387) | (299) |
| Maintenance stage for Diabetes | 644 | -1,103 | 190 | -1,704** |
|  | (852) | (666) | (423) | (550) |
| Maintenance stage for Heart disease | 1,722* | -498 | 897* | -509 |
|  | (777) | (518) | (380) | (603) |
| Maintenance stage for Hypertension | 503 | -292 | 859** | 322 |
|  | (527) | (321) | (322) | (443) |
| Maintenance stage for Lung disease | 3,889*** | 849** | 491* | 388 |
|  | (458) | (277) | (238) | (283) |
| Maintenance stage for Stroke | 4,046** | 694 | 1,959* | 950 |
|  | (1,434) | (1,058) | (819) | (780) |
| Maintenance stage for Heart attack | 1,036 | 719 | 330 | -234 |
|  | (705) | (434) | (347) | (290) |
| Maintenance stage for Dementia | 771 | 549 | -826* | 811 |
|  | (907) | (647) | (406) | (445) |
| Living in nursing home | 69,351*** | 8,031*** | 4,815*** | 45,313*** |
|  | (2,897) | (2,324) | (868) | (2,541) |
| ADL 3+, Not in nursing home | 11,507*** | 4,993*** | 2,577*** | 707** |
|  | (876) | (622) | (394) | (229) |
| Eligible for Medicare due to disablity | -3,591 | -4,411 | -3,724 | -939 |
|  | (2,683) | (2,676) | (2,997) | (1,477) |
| Died | 41,590*** | 19,685* | 7,680 | 4,153 |
|  | (10,733) | (9,651) | (4,079) | (3,020) |
| Diabetes × Heart disease | 2,941*** | 1,374* | 529 | 1,819*** |
|  | (864) | (592) | (421) | (482) |
| Diabetes × Hypertension | 3,391*** | 1,342* | 189 | 763 |
|  | (940) | (672) | (477) | (623) |
| Hypertension × Heart disease | 1,983* | 1,191* | 373 | -299 |
|  | (874) | (593) | (452) | (635) |
| Hypertension × Stroke | -712 | 882 | -1,137 | -448 |
|  | (1,616) | (1,277) | (830) | (856) |
| Disability claim × Nursing home | -585 | 6,549 | -414 | -2,971 |
|  | (10,541) | (6,260) | (1,597) | (5,829) |
| Died × Nursing home | -52,881*** | -15,679*** | -7,387*** | -23,924*** |
|  | (3,549) | (2,416) | (966) | (3,316) |
| Died × age 65-69 | -1,160 | 10,518 | 3,831 | -5,797 |
|  | (14,392) | (11,926) | (5,717) | (3,663) |
| Died × age 70-74 | -15,982 | 8,434 | 125 | -2,537 |
|  | (12,288) | (12,839) | (4,534) | (3,414) |
| Died × age 75-79 | -8,852 | 12,605 | 962 | -3,515 |
|  | (12,176) | (12,374) | (4,708) | (3,930) |
| Died × age 80-84 | -10,258 | 5,869 | -396 | -1,017 |
|  | (11,264) | (10,153) | (4,305) | (4,036) |
| Died × age 85+ | -20,391 | -765 | -2,593 | -3,680 |
|  | (10,922) | (9,855) | (4,107) | (3,850) |
| Nursing home × Dementia | 5,886 | 1,134 | -2,021* | 6,370* |
|  | (3,340) | (2,775) | (858) | (2,556) |
| Nursing home × Heart attack | -6,259 | -288 | -1,539 | -4,570 |
|  | (3,639) | (2,365) | (1,327) | (3,008) |
| Intercept | 15,256*** | 4,238 | 6,591* | 3,087 |
|  | (2,587) | (2,653) | (2,934) | (1,595) |
| R^2^ | 0.212 | 0.163 | 0.069 | 0.594 |
| N | 29,465 | 17,606 | 16,909 | 4,938 |

Notes: OLS regression models of annual medical costs estimated using Medicare Current Beneficiary Survey (MCBS) data, 2016–2019. Costs are reported in 2019 US dollars. Separate models are estimated for total medical costs, Medicare Part A, Medicare Part B, and Medicaid spending. Chronic conditions enter the model in two stages: a diagnosis stage indicator for the year of initial diagnosis and a maintenance stage indicator for subsequent years. Interaction terms capture the combined effects of comorbid conditions and of nursing home residence and death with other covariates. The reference age category is under 65. Standard errors are in parentheses. *p < 0.05, **p < 0.01, ***p < 0.001.

### Table A6. Health-related quality of life model

|  | Health-related quality of life (0-100) |
| --- | --- |
| CDR-SB | -2.278*** |
|  | (0.464) |
| Cancer | 0.939 |
|  | (2.282) |
| Diabetes | -7.825*** |
|  | (2.295) |
| Heart disease | -10.885*** |
|  | (2.012) |
| Hypertension | -3.818* |
|  | (1.657) |
| Lung disease | -13.594*** |
|  | (2.840) |
| Stroke | -14.190*** |
|  | (2.875) |
| Heart attack since last wave | -4.485 |
|  | (5.098) |
| Current smoking | 0.312 |
|  | (2.300) |
| Obese | -0.316 |
|  | (2.012) |
| Single | -3.030 |
|  | (2.569) |
| Widowed | -5.222** |
|  | (1.926) |
| Intercept | 83.211*** |
|  | (1.440) |
|  |  |
| R^2^ | 0.184 |
| N | 897 |

Abbreviations: CDR-SB, Clinical Dementia Rating – Sum of Boxes; HUI3, Health Utilities Index Mark 3.

Notes: OLS regression model of health-related quality of life measured using the Health Utilities Index Mark 3 (HUI3). We rescale the HUI3 utility value back to a visual analogue scale form based on equations in Feeny et al. (2002); the outcome therefore ranges 0 to 100. Estimated using HRS data from the 2000 wave, which included the HUI3 instrument in an experimental module. The dependent variable ranges from 0 (equivalent to death) to 100 (perfect health); negative values are possible on the original HUI3 scale but the model is estimated on the 0–100 rescaled version. Standard errors are in parentheses. *p < 0.05, **p < 0.01, ***p < 0.001.

### Table A7. Health-related quality of life using alternative methods

|  | Lifetime value of health-related quality-of-life gain  per person (2025 USD) | |
| --- | --- | --- |
|  | GRACE | HUI3 |
| Treatment | 52,339 | 47,712 |
|  | (50,313, 54,365) | (45,869, 49,555) |
| Earlier Treatment | 72,714 | 66,719 |
|  | (69,729, 75,699) | (64,079, 69,359) |

Notes: Lifetime value of the gain in health-related quality of life under each treatment scenario, discounted at 3%. The GRACE column applies the Generalized Risk-Adjusted Cost-Effectiveness framework used in the main analysis (the quality-of-life values reported in Table 3); the HUI3 column values the same simulated changes in HUI3 utility at a constant willingness-to-pay. Bootstrapped 95% confidence intervals are in parentheses.

### Table A8. Range of gross societal value of treatment accounting for trial-reported uncertainty

|  | Lifetime value per person (2025 USD) | |
| --- | --- | --- |
|  | Treatment | Earlier Treatment |
| Rate of slowing (%) |  |  |
| 18.3 | 61,616 | 81,419 |
| 28.9 | 104,885 | 138,256 |
| 39.5 | 153,801 | 201,770 |

Notes: Gross societal value per person of each treatment scenario when the rate of CDR-SB slowing is set to the point estimate (28.9%) and to the bounds of its 95% confidence interval (18.3%, 39.5%) for the combined population in TRAILBLAZER-ALZ 2. The 28.9% row corresponds to the main-analysis estimates reported in the text.

# Appendix B. Imputation of CDR-SB in the HRS

The HRS core survey does not include the Clinical Dementia Rating – Sum of Boxes (CDR-SB). However, CDR-SB was assessed in the Aging, Demographics, and Memory Study (ADAMS), a sub-study of the HRS in which clinical assessments for dementia were conducted for a subsample of HRS respondents. ADAMS Wave A was conducted in 2001–2003, and included 856 respondents with non-missing CDR-SB scores. We used the ADAMS sample to impute CDR-SB in the broader HRS sample through a nearest-neighbor matching procedure.

We first linked each ADAMS respondent to their closest HRS core interview. ADAMS interviews took place between the 2000 (wave 5) and 2002 (wave 6) core HRS interviews. Each ADAMS respondent was assigned to the most recent core wave for which they had a completed interview at or before the ADAMS interview date, ensuring that the core HRS data preceded or coincided with the ADAMS clinical assessment.

We then matched each core HRS respondent-wave observation (age 58 and older) to an ADAMS respondent using the Stata command kmatch (Jann, 2017), which implements Mahalanobis distance nearest-neighbor matching. ADAMS respondents served as donors and were matched with replacement, allowing a single ADAMS respondent to serve as the donor for multiple HRS observations. Matching variables included standardized versions of the TICS-27 total cognition score, number of ADL limitations, number of IADL limitations, years of education, and sex. Respondents were exactly matched on cognitive status category (normal, cognitive impairment not dementia, or dementia, based on the Langa-Weir classification), age 80 or older, and proxy interview status. After matching, each HRS respondent was assigned the CDR-SB and global CDR score of their matched ADAMS donor.

Observations with missing values on any matching variable were excluded from the procedure. The resulting imputed sample covers HRS waves 5 through 16 (2000–2022) for respondents aged 58 and older.

Figure B1 compares the distribution of CDR-SB in the ADAMS sample (observed) and the HRS sample (imputed), restricting the HRS to the 2000 and 2002 waves and respondents aged 70 and older to approximate the timing and age range of the ADAMS sample. Both distributions are weighted using their respective survey weights. The imputed distribution closely tracks the observed ADAMS distribution, providing evidence that the matching procedure preserves the population-level distribution of CDR-SB.

### Figure B1. Distribution of CDR-SB in imputed and observed samples

Notes: Kernel density plots weighted using ADAMS and HRS survey weights. CDR-SB is observed in ADAMS and imputed in HRS. The HRS sample is restricted to the 2000 and 2002 waves to match the timing of the ADAMS sample.

# Appendix C. Construction of analysis cohort

## Sample Selection

We constructed the USCDM analysis cohort by selecting HRS respondents meeting the following criteria, designed to approximate the TRAILBLAZER-ALZ 2 clinical trial eligibility requirements:

1. Aged 60–85 at the time of interview.
2. Alive at interview with nonzero survey weights.
3. Global CDR score of 0.5 (MCI) or 1 (mild dementia), based on imputed CDR (Appendix B).
4. Non-missing CDR-SB at both the current and two-year prior interview wave, with CDR global greater than zero two years earlier (indicating that cognitive symptoms were present before the current observation).
5. No history of non-skin cancer within the past five years, consistent with the trial's exclusion of participants with recent serious illness.
6. TICS-27 total cognition score of 16 or below, corresponding approximately to an MMSE score of 20–28 based on a published crosswalk, consistent with the trial's cognitive eligibility range.

To increase the pool of potentially eligible respondents resembling clinical trial participants, we pooled HRS observations from 2010 to 2020. For respondents appearing in multiple eligible waves, we retained their most recent eligible observation. We also restricted the sample to one respondent per household to avoid computational issues in the simulation. The resulting cohort included 6,339 HRS respondents. For each respondent in the main USCDM cohort, we retrieved their HRS data from the preceding wave (one wave earlier, corresponding to approximately two years prior). This cohort entered the simulation at t = −2 with their observed characteristics at that earlier time point, including a lower mean CDR-SB of 2.3. The same reweighting procedure and weights from the main cohort were applied.

## Reweighting

Clinical trial participants were highly selected on cognitive status, education, and race relative to the general population of individuals with early symptomatic AD. Moreover, the HRS did not include information on amyloid pathology, so we could not directly select for amyloid positivity as required by the trial. We therefore reweighted the USCDM cohort to match the baseline characteristics of the TRAILBLAZER-ALZ 2 combined tau population (i.e., using HRS respondents’ characteristics in t = 0).

We used the sreweight command in Stata (Pacifico, 2014), which implements the methodology of Deville and Särndal (1992) for calibrating survey weights to match known population totals. This approach adjusts existing survey weights as little as possible (in a distance-minimizing sense) while ensuring that the reweighted sample moments match specified targets. We used a modified chi-squared distance function with an upper bound of 20 on individual weights and a lower bound near zero.

The reweighting targets were derived from the baseline characteristics reported for the TRAILBLAZER-ALZ 2 combined tau population. The following variables were included as reweighting targets:

- the proportion aged 60–64, 65–74, and 75–85;
- the proportion male;
- the proportion non-Hispanic White;
- the proportion with 13 or more years of education;
- the proportion with a global CDR of 0.5;
- mean CDR-SB; and
- mean CDR-SB two years earlier (estimated from the matched ADNI cohort described in the text).

CDR-SB variables were rescaled (divided by 10) to improve numerical stability in the optimization.

After reweighting, 45% of observations had weights between 0.05 and 20, and 55% had weights below 0.05. The reweighted USCDM cohort closely matched the trial population on all targeted characteristics (Table 1 in the text). These rebalancing weights were used in all subsequent analyses so that results are representative of the clinical trial participant population.
